# Age-Related Inter-Region EEG Coupling Changes During the Control of Bottom–Up and Top–Down Attention

**DOI:** 10.3389/fnagi.2015.00223

**Published:** 2015-12-01

**Authors:** Ling Li, Dandan Zhao

**Affiliations:** Key Laboratory for NeuroInformation of Ministry of Education, School of Life Science and Technology, University of Electronic Science and Technology of ChinaChengdu, China

**Keywords:** aging, electroencephalographic (EEG), visual pop-out, visual search, control of attention, inter-region phase coupling, theta, alpha

## Abstract

We investigated age-related changes in electroencephalographic (EEG) coupling of theta-, alpha-, and beta-frequency bands during bottom–up and top–down attention. Arrays were presented with either automatic “pop-out” (bottom–up) or effortful “search” (top–down) behavior to younger and older participants. The phase-locking value was used to estimate coupling strength between scalp recordings. Behavioral performance decreased with age, with a greater age-related decline in accuracy for the search than for the pop-out condition. Aging was associated with a declined coupling strength of theta and alpha frequency bands, with a greater age-related decline in whole-brain coupling values for the search than for the pop-out condition. Specifically, prefronto-frontal coupling in theta- and alpha-bands, fronto-parietal and parieto-occipital couplings in beta-band for younger group showed a right hemispheric dominance, which was reduced with aging to compensate for the inhibitory dysfunction. While pop-out target detection was mainly associated with greater parieto-occipital beta-coupling strength compared to search condition regardless of aging. Furthermore, prefronto-frontal coupling in theta-, alpha-, and beta-bands, and parieto-occipital coupling in beta-band functioned as predictors of behavior for both groups. Taken together these findings provide evidence that prefronto-frontal coupling of theta-, alpha-, and beta-bands may serve as a possible basis of aging during visual attention, while parieto-occipital coupling in beta-band could serve for a bottom–up function and be vulnerable to top–down attention control for younger and older groups.

## Introduction

Normal aging is associated with decline in the performance of a variety of cognitive functions, including effects observed in the visual search (Plude and Doussard-Roosevelt, 1989) and in the tasks involving both bottom–up attention and top–down attention, with a more prominent decline in tasks emphasizing top–down attention control (Greenwood et al., 1997; Kok, 2000; Madden et al., 2005; Madden, 2007; Lien et al., 2011; Li et al., 2013). Normal aging is correlated with changes in neural structure, including decline in brain volume (Scahill et al., 2003; Fotenos et al., 2008) and gray and white matter volumes (Raz et al., 2005; Gordon et al., 2008). Age-related losses in gray and white matter in medial-temporal, parietal, and frontal areas were reported. Hypotheses of age-related brain functional changes mainly include Compensation Related Utilization of Neural Circuits Hypothesis (CRUNCH) (Reuter-Lorenz and Lustig, 2005; Grady, 2008; Reuter-Lorenz and Cappell, 2008) and dedifferentiation hypothesis (Madden et al., 2007).

A variety of functional imaging, neurophysiological and neuropsychological studies have provided compelling evidence that frontoparietal networks play important roles in both top–down cognitive and bottom–up sensory factors of attention control (Corbetta and Shulman, 2002; Giesbrecht et al., 2003; Bledowski et al., 2004a,b Buschman and Miller, 2007; Husain and Nachev, 2007; Knudsen, 2007; Li et al., 2010). An aging study of visual attention has reported that older adults showed increased magnitude and spread of activity in fronto-parietal regions compared with younger adults, suggesting a compensation for a decline in overall bottom–up sensory input (dedifferentiation; Madden et al., 2007; see reviews Reuter-Lorenz and Park, 2010). However, activity decreases with aging in frontal cortex (Anderson et al., 2000; Milham et al., 2002; Johnson et al., 2004) and occipital cortex (Madden et al., 2004) were also reported in attention studies, pointing to an age-related decline in allocation of attentional resources efficiency (Lorenzo-López et al., 2008) or reduction in inhibitory control functions in attention (Colcombe et al., 2003; Madden et al., 2004; Andrés et al., 2006; Hasher et al., 2008). Performance has been found associated with fronto-parietal activation for older adults and with occipital activation for younger adults in top–down attention (Madden et al., 2007), but with prefrontal activation for younger adults and with deep gray matter structures for older adults in visual target detection (Madden et al., 2004). Increased activities in frontal regions associated with improved performance (Grady et al., 2002; Madden et al., 2004; Lorenzo-López et al., 2008; Vallesi et al., 2010), or with decreased performance (Madden et al., 2005) in older adults have also been reported in certain tasks. The CRUNCH interprets these contradictory results at some degree, whereby older adults use more or new neural circuits to accomplish tasks compared to younger adults.

A functional imaging study shows that aging is associated with decreased connectivity between areas within the fronto-parietal control network and between areas within the somatomotor network in a selective attention task, but with increased connectivity between the fronto-parietal and somatomotor network (Geerligs et al., 2014). Functional connectivity decreases with aging within the fronto-parietal regions during cue processing under executive control was reported (Madden et al., 2010). There has been study showing both selective increases between visual attention regions and supplementary motor area and decreases between sensorimotor systems and supplementary motor area in resting-state functional connectivity with age (Roski et al., 2013). In summary, aging is associated with lower connectivity within task-relevant networks and greater connectivity between the task-relevant networks and outside networks by functional imaging studies (Madden et al., 2005, 2010; Dennis et al., 2008; St Jacques et al., 2009; Geerligs et al., 2014), supporting the CRUNCH that older adults use more or new neural circuits to compensate for age-related decline.

In electrocephalogram (EEG) studies, older adults show reduced frontal theta activity during sustained attentional processes, and reduced theta connectivity strength within frontal regions and between frontal midline and temporal cortices during working memory maintenance (Kardos et al., 2014). Decreased posterior alpha activation in an attention network test (Deiber et al., 2013) and reduced beta activation in a visual attentional task correlated to alertness and sustaining attentional processes (Gola et al., 2012, 2013) with aging were reported. Aging is associated with decreased modularity and clustering and increased connectedness of anterior nodes in beta-band network during resting condition, pointing to a compensation of the anterior atentional system (Knyazev et al., 2015). These findings provide evidence that aging modulates distinct neural circuits at different oscillatory frequencies during attention functions. There is, however, relatively little evidence directly investigating aging-related functional connectivity by EEG in bottom–up and top–down attention together.

We used a paradigm based on a study in non-human primates by Buschman and Miller (2007) to investigate age-related coupling of different frequency bands during top-down and bottom-up attention control. Arrays were presented with either automatic “pop-out” (bottom–up) or effortful “search” (top–down) behavior to younger and older participants. Buschman and Miller found that fronto-extrastriate coherences were greater in the search than in the pop-out condition at low gamma-band (22–34 Hz) and parietal–extrastriate coherences were greater in the pop-out than in the search condition at high gamma-band (35–55 Hz). In a human EEG study, a double dissociation was reported, with significantly increased power from 4 to 24 Hz in parietal areas for pop-out targets and increased power from 4 to 24 Hz in frontal regions for search targets (Li et al., 2010). Greater frontal-parietal synchrony at low gamma-band frequencies for inefficient than efficient visual search were reported (Phillips and Takeda, 2009). Based on above neuroimaging and electrophysiology literatures, as well as the results from our previous studies (Li et al., 2010, 2013), we expected the differential roles of fronto-parieto-occipital connectivity at different lower oscillatory frequencies under both types of attention for younger and older groups. The purpose of the present study was to examine the effects of aging on functional connectivity at different oscillatory frequencies during visual search and simultaneously compare the fronto-parieto-occipital connectivity during top–down and bottom–up attention. We hypothesized that (a) aging and search condition are associated with decreases in connectivity due to a slowing in performance, and (b) connectivity at different oscillatory frequencies plays a differential role contributing to group and attentional control.

## Materials and Methods

### Subjects

We utilized data reported in a previous study (Li et al., 2013) consisting of 13 younger subjects (6 females, mean age ± standard deviation = 23.9 ± 4.3 years, range from 18 to 35 years old) and 13 older subjects (6 females, mean age ± standard deviation = 63.1 ± 6.2 years, range from 52 to 75 years old). The mean numbers of years of education for the younger and older subjects were 15.9 ± 2.5 and 16.0 ± 2.2 years respectively. All the subjects were right-handed, had normal color vision, and had no history of neurological problems. None of the subjects were taking any psychotropic, neurological, or psychiatric medications at the time of testing. The experimental procedures were approved by the Committee for the Protection of Human Subjects for the University of California, Berkeley. Written Informed consent was obtained from all subjects prior to being tested.

### Stimuli and Procedure

**FIGURE 1:**
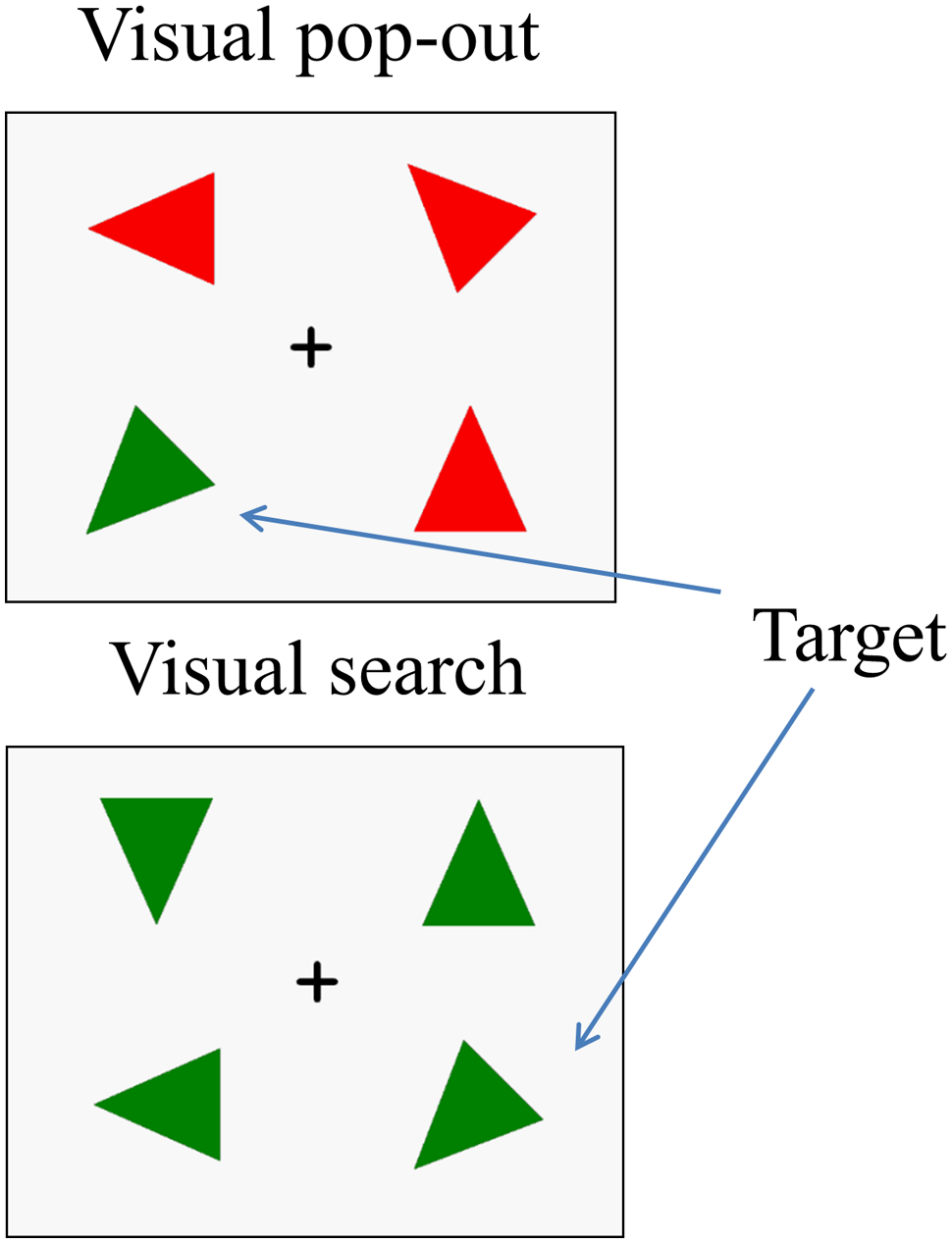
**An example of the stimulus array in the two experimental conditions.** Each trial includes a target. In the **(up panel)**, the distractors differ from the target in both color and orientation, so that target detection is highly efficient and easy. In this pop-out condition, search is influenced primarily by the bottom–up attention. In the **(down panel)**, the distractors differ from the target only in orientation, so that target detection is less efficient and difficult. In this search condition, target detection is controlled mainly by top–down attention.

The stimuli were made up of 16 acute isosceles triangles, each with a particular color (red or green) and orientation [one of eight, (i-1) × 45°, *i* = 1, 2, 3, 4, 5, 6, 7, 8]. The triangles had two equal sides 6.5 cm in length and a third side 5.5 cm long, with an area of 16.20 cm × 16.20 cm. **Figure 1** illustrates an example of the stimulus sequence. After a 500 ms fixation cross, a target triangle (one of 16 triangles) appeared in the center of the screen for 1000 ms and was followed by a short 500 ms delay screen with a fixation cross. After the delay, a four stimulus array was presented, consisting of the target and three distracter triangles in the four quadrants of the screen. The target was randomly presented in one of these locations (upper-left, lower-left, upper-right, and lower-right). The center of each triangle was 6.2 cm vertical (either up or down) from the horizontal midline and 8.2 cm lateral (either right or left) from the vertical midline, resulting in stimuli at a visual angle of 5.34° from fixation. The array remained on the screen until a response and was followed by a 1000 ms fixation to show the end of the trial. Three distractor triangles were chosen to create the two main attention conditions in the experiment: “pop-out” and “search.” The pop-out condition was created using distracters that differed from the target in both color and orientation (Treisman and Gelade, 1980), while the search condition was created by using distracters that differed from the target only in orientation. Half of the trials were in the pop-out condition and half were in the search condition. Half of the targets were presented in left visual field and half were in right visual field.

Subjects were asked to centrally fixate throughout the recording and to respond as quickly as possible whether the target was to the left or right of fixation. Participants used their right-hand for responding by pressing either button 1 for left or 2 for right from a computer key pad. Participants performed two practice blocks before starting the experiment and extra practice blocks were given as required until subjects were able to reach a mean accuracy of 80% in the task. After the practice, 12 experimental blocks comprising 32 trials each lasting about 2.5 min were run. There were 1–2 min breaks between blocks, with longer breaks every three blocks. E-prime (Psychology Software Tools, Pittsburgh, PA, USA) was used to present the stimuli and analyze the behavioral data.

### Data Recording and Preprocessing

EEG was recorded by an ActiveTwo system (Biosemi, The Netherlands) with a 64 channel electrode cap. Right and left earlobes and four electrooculogram (EOG) were simultaneously recorded. EEG data were off-line referenced to the average of the right and left earlobes. EOG was measured from an electrode above and below the right eye to record vertical eye movements and electrodes on the outer canthus of each eye to measure horizontal eye movements. All channels were amplified with an analog bandpass filter of 0.06–208 Hz and were digitized at 1024 Hz.

Matlab was used for all data processing. Re-referenced EEG signals were filtered from 0.5 to 55 Hz with a two-way FIR bandpass filter (eegfilt.m from EEGLAB toolbox, Delorme and Makeig, 2004) and segmented from 200 ms before the onset of the stimulus (visual array) to 1000 ms after the stimulus. Trials were rejected if they had an incorrect response or lacked a button press between 200 and 1500 ms (younger adults) or 200–2000 ms (older adults) after the onset of the stimulus array. Epochs with EOG artifacts were removed if there was a difference in amplitude between the two vertical EOG or between the two horizontal EOG of greater than 100 μV, or if there was more than 3 standard deviations (SDs) from the mean of the EOG difference wave. For each epoch, the linear drift was removed and the data was baseline corrected using the 200 ms pre-stimulus period. Finally, any epoch with a channel containing amplitudes of more than four standard deviations from the epoch mean was rejected. After above preprocessing, 537 trials remained for left pop-out targets, 480 trials remained for right pop-out targets, 479 trials remained for left visual search targets, and 424 trials remained for right search targets for the younger participants. 417 trials remained for left pop-out targets, 402 trials remained for right pop-out targets, 332 trials remained for left visual search targets, and 344 trials remained for right search targets for the older participants. These trials were used for ERP analysis (see in Li et al., 2010, 2013) and for the current EEG coupling analysis. At least 25 trials were included in the average for each condition.

### EEG Cross-channel Coupling Analysis

To minimize the contribution of volume conduction and remove spurious coupling (Nunez et al., 1997; Lachaux et al., 1999), the following steps were applied to single trial of EEG data before the computation of the phase synchrony. Step 1: each single trial of 4 conditions (2 × 2, target condition and visual field) of every subject for both groups was filtered by the band-pass finite impulse response filters at 4 Hz intervals between 4 and 40 Hz. Totally signals of 9 frequency bands EEG were obtained. Step 2: we used a current source density (CSD) toolbox of MATLAB supplied by Kayser J. (http://psychophysiology.cpmc.columbia.edu/Software/CSDtoolbox/index.html) that implemented a spherical spline algorithm of Perrin et al. to estimate scalp current density (SCD) for EEG data (Perrin et al., 1989; Kayser and Tenke, 2006). The spline interpolation constant was set to 4.

After above SCD computation, the data from 200 ms before the onset of stimuli array to 1000 ms after the stimuli were used to estimate long-range neural phase synchrony in nine frequency bands by calculating phase-locking value (PLV). The PLV has been used to measure the bivariate phase synchronization in a number of EEG studies (Mormann et al., 2000; Quian Quiroga et al., 2002; Knyazev et al., 2015). The PLV between electrodes *j* and *k*, at each sample time *t*, across the *N* trials, were quantified as _
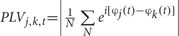
_. Instantaneous phase φ(*t*) of a signal was estimated by Hilbert transform.

The PLV takes on values between 0 and 1, but the PLV must first be normalized before it can be used as a metric of cross-electrode coupling strength. That is, we are interested in the properties of the distribution of phase difference between two electrodes. One way is to compare the actual mean PLV with a set of surrogate PLV created by offsetting phase of one signal by some large time lag. Two hundred surrogate PLV values for each frequency and time point were calculated, and the mean and standard deviation of surrogate PLV were estimated. Therefore, the normalized PLV [(real PLV – mean of surrogate PLV)/standard deviation of surrogate PLV] were defined as the modulation index used in this paper. For a given number of sample points, we can directly compare this normalized PLV for cross-electrode coupling strength across different pairs as well as different frequency bands which may have very different power levels. The normalized PLV index was computed for all pair-wise combinations of channels, generating 2016 (totally 64 channels) index values for each time point in 9 frequency bands in each condition for both groups. With α = 0.01 and *N* = 2016 comparisons, an index value greater than 4.42 was required for significance by Bonferroni correction for multiple comparisons.

### Statistical Analysis

Behavioral data were analyzed with a repeated measure ANOVA with condition (pop-out and search) and target visual field (left and right) as the within-subject factors and age (young and old) as the between-subject factor, followed by Bonferroni corrected *t*-tests if necessary with *p*-value <0.05 as a significant threshold.

Mean normalized PLV values and mean numbers of connection were calculated first by the mean of whole-brain significant coupling values in 0–600 ms time-window. Then mean normalized PLV multiplied by mean numbers of connection to obtain total coupling values. Finally, the total coupling values were analyzed with a repeated measure ANOVA with condition (pop-out and search) and target visual field (left and right) as the within-subject factors and age (young and old) as the between-subject factor for 9 frequency bands.

**FIGURE 2:**
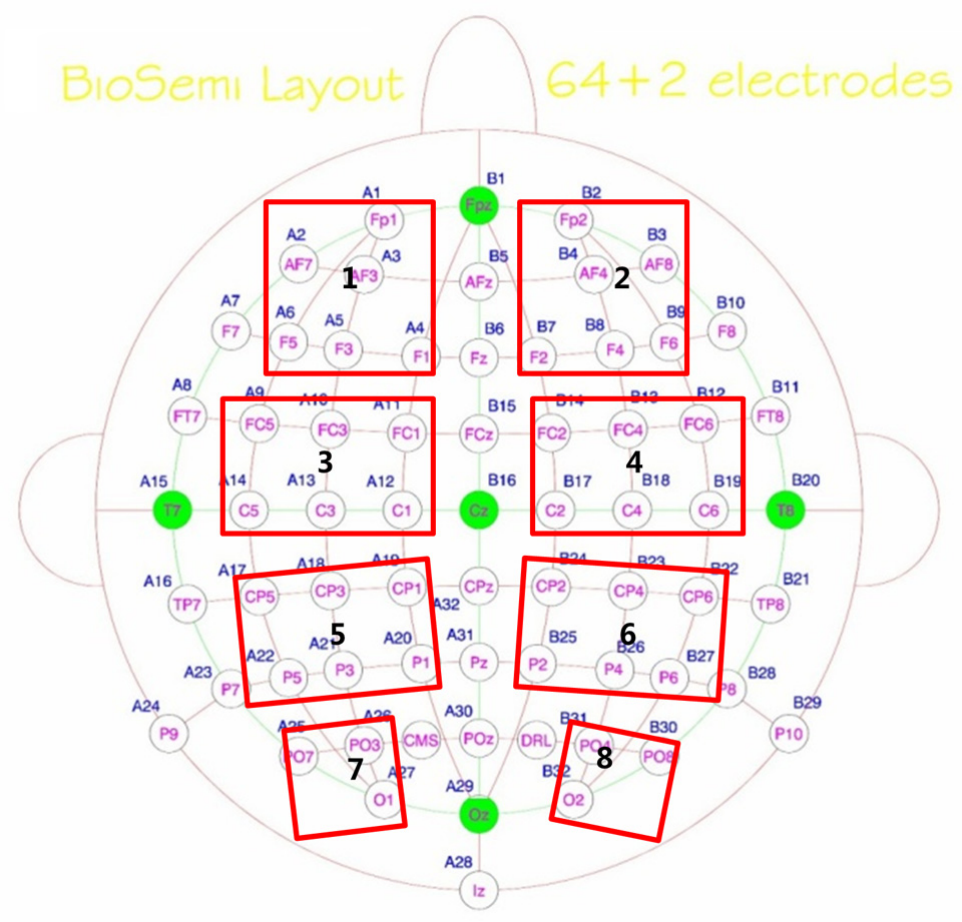
**Eight regions of interest (ROIs, marked by red rectangles) for inter-region analysis were selected including left prefrontal (Fp1, AF7, AF3, F5, F3, F1), right prefrontal (Fp2, AF8, AF4, F6, F4, F2), Left central-frontal (FC5, FC3, FC1, C5, C3, C1), right central-frontal (FC6, FC4, FC2, C6, C4, C2), Left central-parietal (CP5, CP3, CP1, P5, P3, P1), right central-parietal (CP6, CP4, CP2, P6, P4, P2), Left parietal-occipital (PO7, PO3, O1), and right parieto-occipital (PO8, PO4, O2) regions**.

Eight regions of interest (ROIs) were selected including left prefrontal (Fp1, AF7, AF3, F5, F3, F1), right prefrontal (Fp2, AF8, AF4, F6, F4, F2), left central-frontal (FC5, FC3, FC1, C5, C3, C1), right central-frontal (FC6, FC4, FC2, C6, C4, C2), left central-parietal (CP5, CP3, CP1, P5, P3, P1), right central-parietal (CP6, CP4, CP2, P6, P4, P2), left parietal-occipital (PO7, PO3, O1), and right parieto-occipital (PO8, PO4, O2) regions (see **Figure 2**). Max normalized PLV values for all pair-wise combinations of eight regions of interest were calculated, generating 28 (totally 8 ROIs) index values for frequency bands of interest in four conditions for both groups. With α = 0.05 and *N* = 28 comparisons, an index value greater than 2.91 was required for significance by Bonferroni correction for multiple comparisons. Each significant connectivity between ROIs were then analyzed with a repeated measure ANOVA with condition (pop-out and search) and target visual field (left and right) as the within-subject factors and age (young and old) as the between-subject factor for frequency bands of interest.

Linear relationship between above coupling values and behaviors were then tested by Pearson correlation coefficient for four conditions in both groups in each frequency band, respectively.

## Results

### Behavioral Results

Mean reaction times (RTs) and accuracy rates are summarized in **Table 1** (mean ± standard deviation). There was a main effect of age (young and old), condition (pop-out and search), and target visual field (left and right) on mean RTs [age effect: *F*(1,24) = 31.35, *p* < 0.001; condition effect: *F*(1,24) = 786.05, *p* < 0.001; target visual field effect: *F*(1,24) = 5.70, *p* = 0.025, ANOVA], with slower RTs in the older subjects and in the search condition and in the left visual field target. However, there was no significant interaction in RTs among age, condition and target visual field. A main effect of age and condition was observed on accuracy rates [age effect: *F*(1,24) = 19.75, *p* < 0.001; condition effect: *F*(1,24) = 88.55, *p* < 0.001], with higher accuracy overall in the younger group and in the pop-out condition. There was a significant interaction in accuracy between age and condition [*F*(1,24) = 14.28, *p* = 0.001], showing an increased decline in accuracy for the older compared with the younger subjects in the search condition compared with the pop-out condition.

**Table 1:** Behavioral results (mean ± SD).

Condition	Target visual field	RT (ms)		Accuracy (%)	
		Young	Old	Young	Old
Visual pop-out	Left	487.74 ± 106.82	704.91 ± 97.57	99.03 ± 1.16	97.75 ± 1.64
	Right	476.44 ± 94.39	678.11 ± 96.20	99.20 ± 0.87	98.16 ± 1.60
Visual search	Left	775.67 ± 129.86	1025.53 ± 74.26	92.74 ± 4.75	81.15 ± 9.35
	Right	768.45 ± 106.63	979.53 ± 148.64	93.13 ± 3.88	85.80 ± 7.64

*Mean reaction times (RT, ms) and accurate rates (%) and their corresponding standard deviations as a function of condition and target visual field in younger and older subjects.*

Reaction times and accuracy rates were assessed using a two-way repeated ANOVA with condition and target visual field for both groups, respectively. There was only a main effect of condition on mean RTs and accuracy rates for younger group [RTs: *F*(1,12) = 449.51, *p* < 0.0001; accuracy rates: *F*(1,12) = 30.95, *p* < 0.001], with quicker RTs and higher accuracy in the pop-out condition. For older group, there was a main effect of condition on mean RTs and accuracy rates [RTs: *F*(1,12) = 355.06, *p* < 0.0001; accuracy rates: *F*(1,12) = 58.46, *p* < 0.001], with quicker RTs and higher accuracy in the pop-out condition, and there was a main effect of target visual field only on RTs [*F*(1,12) = 5.06, *p* = 0.044], with slower RTs in the left visual field target.

### EEG Coupling Results

#### Whole-brain Total Coupling Values

Supplementary Table S1 summarizes the statistical effects of three factors of age, condition, and target visual field and interaction on the total coupling values for 9 frequency bands. As can be seen in Supplementary Table S1, there was a main effect of age for total coupling values only in the theta and alpha bands [theta band: *F*(1,24) = 9.28, *p* = 0.006; alpha band: *F*(1,24) = 5.89, *p* = 0.023], and there was a main effect of condition in almost all frequency bands. Since there was not any effect of age or any interaction effect between age and other factors in the higher frequency bands (24–40 Hz), we did not report these results in the following analyses. In three beta bands (12–24 Hz) there was an interaction between age and target visual field, so that we averaged their coupling values as one beta band to analyze. Hence, the frequency bands of interest were theta (4–8 Hz), alpha (8–12 Hz), and beta (12–24 Hz) activities.

**Table 2** presents the total coupling values of whole-brain in the theta, alpha, and beta frequency bands of two target visual fields for the pop-out and search conditions in younger and older participants. For theta frequency band, there was a main effect of age (young and old) and target visual field (left and right), with bigger coupling values in the younger subjects only for search condition [independent *t*-tests, pop-out and left: *t*_(24)_ = 2.39, Bonferroni corrected *p* > 0.05; pop-out and right: *t*_(24)_ = 2.23, corrected *p* > 0.05; search and left: *t*_(24)_ = 3.57, corrected *p* < 0.05; search and right: *t*_(24)_ = 2.79, corrected *p* < 0.05]. There was an interaction between age and visual target field [*F*(1,24) = 6.50, *p* = 0.018], so that we tested two-way repeated measure ANOVA for the total coupling values with condition (pop-out and search) and target visual field (left and right) as the within-subject factors for both groups, respectively. There was only a main effect of target visual field for younger group [*F*(1,24) = 9.26, *p* = 0.01], with bigger coupling values in the left visual field target for pop-out condition [Paired *t*-tests, pop-out: *t*_(12)_ = 2.74, corrected *p* < 0.05; search: *t*_(12)_ = 2.18, corrected *p* > 0.05], but no such effect was present for the elderly group.

**Table 2:** The total coupling values for all pair-wise combinations of electrodes in the theta, alpha, and beta frequency bands (mean ± SEM).

Condition	Target visual field	Theta band (4–8 Hz)		Alpha band (8–12 Hz)		Beta band (12–24 Hz)	
		Young	Old	Young	Old	Young	Old
Pop-out	Left	488.98 ± 64.69	270.28 ± 64.69	724.80 ± 130.16	391.14 ± 130.16	586.45 ± 81.01	433.29 ± 81.01
	Right	422.78 ± 55.43	248.36 ± 55.43	712.57 ± 137.41	375.45 ± 137.41	503.33 ± 74.08	410.13 ± 74.08
Search	Left	445.81 ± 52.71	179.77 ± 52.71	599.18 ± 70.38	249.36 ± 70.38	489.66 ± 56.17	277.76 ± 56.17
	Right	369.09 ± 43.39	198.13 ± 43.39	501.36 ± 60.03	278.36 ± 60.03	398.02 ± 54.24	316.03 ± 54.24

*The total coupling values and their corresponding standard error of mean as a function of condition and target visual field in younger and older subjects.*

For alpha frequency band, there was a main effect of age and a marginal effect of condition on the total coupling values (Supplementary Table S1), and there was no significant interaction among age, condition and target visual field. The total coupling values were smaller for older compared to younger adults only in search condition [independent *t*-tests, pop-out and left: *t*_(24)_ = 1.81, corrected *p* > 0.05; pop-out and right: *t*_(24)_ = 1.74, corrected *p* > 0.05; search and left: *t*_(24)_ = 3.52, corrected *p* < 0.05; search and right: *t*_(24)_ = 2.63, corrected *p* < 0.05].

For beta frequency band, there was a main effect of condition [*F*(1,24) = 7.10, *p* = 0.014] and target visual field [*F*(1,24) = 4.52, *p* = 0.044], but no main effect was present in age. There was an interaction between age and visual target field [*F*(1,24) = 6.40, *p* = 0.018], so that we tested two-way repeated measure ANOVA for the total coupling values with condition (pop-out and search) and target visual field (left and right) as the within-subject factors for both groups, respectively. There was only a main effect of target visual field for younger group [*F*(1,24) = 9.20, *p* = 0.01], with bigger coupling values in the left visual field target for search condition [Paried *t*-tests, pop-out: *t*_(12)_ = 2.23, corrected *p* > 0.05; search: *t*_(12)_ = 2.40, corrected *p* < 0.05], but no such effect was present for the elderly group.

**Supplementary Figure S1** illustrates the presence of significant positive linear relationship within the older adults between total coupling values and RTs in the pop-out condition with left visual field target in theta frequency band [Pearson correlation coefficient (*r*) = 0.588, *p* = 0.035]. A regression was used to test whether a quadratic relationship also contributed to the variance, but it was not significant [*r* = 0.605, *p* = 0.103]. All other correlation between total coupling values and RTs or accuracies was not significant (*p* > 0.05).

#### ROIs Coupling Value in Theta Frequency Band

**FIGURE 3:**
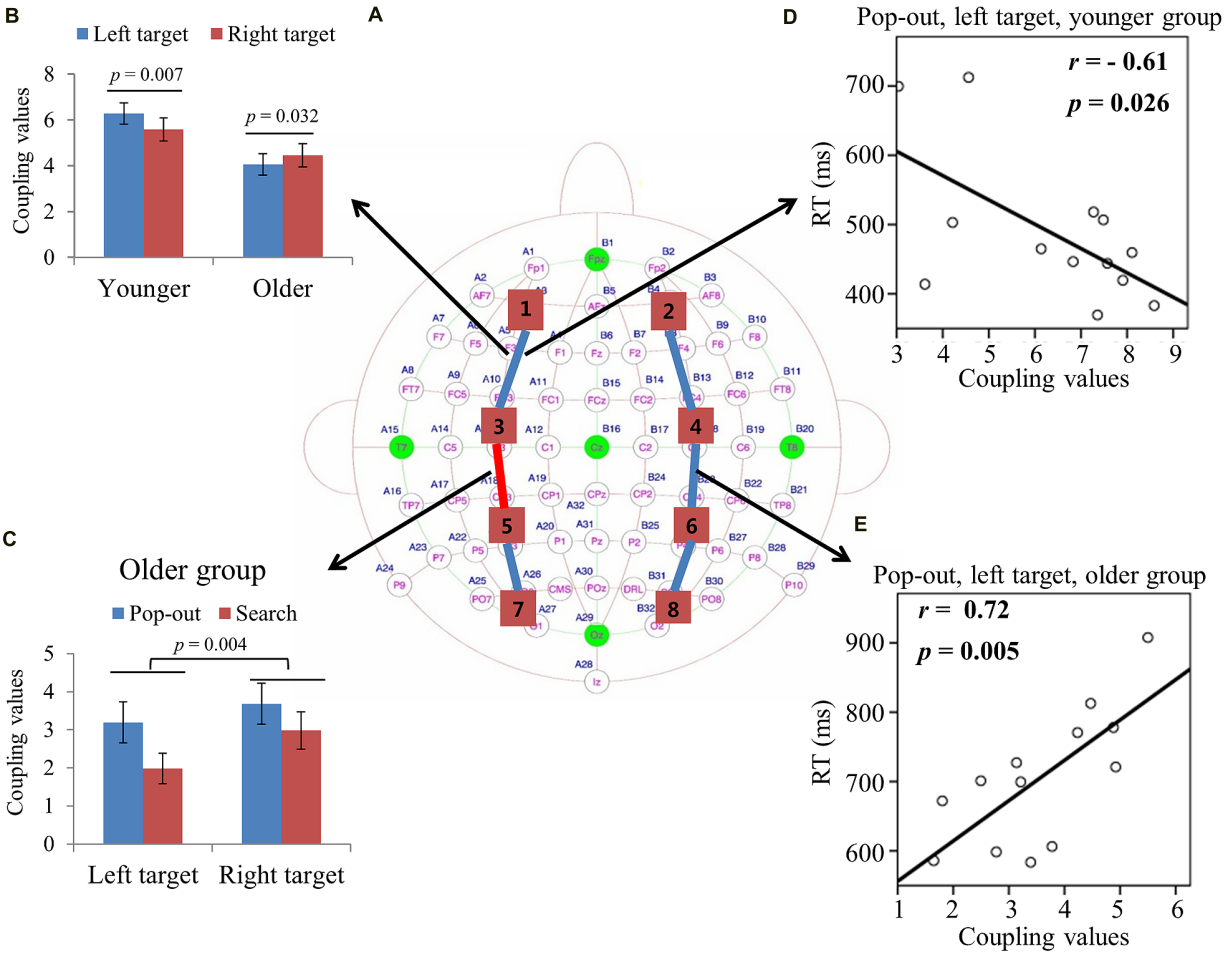
**(A)** Inter-regions coupling in theta frequency band. Blue lines indicate main effect of age (younger > older) and red line indicates main effect of condition (pop-out > search). **(B)** Coupling values between left prefrontal and central-frontal regions as a function of target visual field in younger and older subjects, with greater coupling values in left target than in right target for younger subjects and greater coupling values in right target than in left target for old subjects. **(C)** Coupling values between left central-frontal and central-parietal regions as a function of target visual field in both conditions for older group, with greater values in pop-out condition and in right target, and no such effects for younger group. **(D)** Linear negative regression between mean RT and coupling value between left prefrontal and central-frontal regions for the pop-out condition with the left field target in the younger group. **(E)** Linear positive regression between mean RT and coupling value between right central-frontal and central-parietal regions for the pop-out condition with the left field target in the older group.

Max normalized PLV values for all pair-wise combinations of eight regions of interest were calculated, generating 28 (totally 8 ROIs) index values for theta band (4–8 Hz) in four conditions for both groups. **Figure 3A** summarizes the main effect of age (blue line) and condition (red line) among eight regions of interest. As can be seen in **Figure 3A**, there was a main effect of age for coupling values on five long range connections, including the connections between left prefrontal and left central-frontal regions [1 and 3, *F*(1,24) = 6.14, *p* = 0.02], between right prefrontal and right central-frontal regions [2 and 4, *F*(1,24) = 4.73, *p* = 0.04], between right central-frontal and right central-parietal regions [4 and 6, *F*(1,24) = 5.43, *p* = 0.03], between left central-parietal and left parietal-occipital [5 and 7, *F*(1,24) = 11.47, *p* = 0.002], between right central-parietal and right parieto-occipital regions [6 and 8, *F*(1,24) = 5.60, *p* = 0.04], with smaller coupling values in older subjects. There was a main effect of condition for coupling values between left central-frontal and left central-parietal regions [3 and 5, *F*(1,24) = 6.64, *p* = 0.017], with smaller coupling values in the search condition.

For the connection between left prefrontal and central-frontal regions, there was an interaction between age and target visual field [*F*(1,24) = 7.88, *p* < 0.001], and there was no significant effect of condition. Hence we averaged the coupling values of the pop-out and search conditions, and applied repeated measure ANOVA with target visual field (left and right) as the within-subject factor and age (young and old) as the between-subject factor. **Figure 3B** shows the coupling values in both groups for both target fields. There was a main effect of age [*F*(1,24) = 6.14, *p* = 0.02] and an interaction between age and target field [*F*(1,24) = 16.63, *p* < 0.001], with bigger coupling values in left target than in right target for younger subjects [Paried *t*-tests, *t*_(12)_ = 3.28, *p* = 0.007] and bigger coupling values in right target than in left target for older subjects [Paried *t*-tests, *t*_(12)_ = 2.43, *p* = 0.032].

For the connection between left central-frontal and left central-parietal regions, there was an interaction between age and target visual field [*F*(1,24) = 9.62, *p* = 0.005]. Hence we divided the test into two repeated measure ANOVA with condition and target visual field as the within-subject factors for younger and older groups, respectively. There was a main effect of target field [*F*(1,12) = 12.22, *p* = 0.004] and condition [*F*(1,12) = 5.69, *p* = 0.034] for older group, with greater coupling values in the right field target and in the pop-out condition. There was no significant effect for the younger group. **Figure 3C** shows the coupling values in both conditions and target fields for older group. For the other four long range connections, there was no other significant effect.

**Figure 3D** illustrates the presence of significant negative linear relationship within the younger adults between coupling values of left prefrontal and central-frontal regions and RTs in the pop-out condition with left visual field target [*r* = -0.61, *p* = 0.026]. **Figure 3E** illustrates a significant positive linear relationship within the older adults between coupling values of right central-frontal and central-parietal and RTs in the pop-out condition with left visual field target [*r* = 0.72, *p* = 0.005]. All other correlation between coupling values and RTs or accuracies was not significant (*p* > 0.05).

#### ROIs Coupling Value in Alpha Frequency Band

**FIGURE 4:**
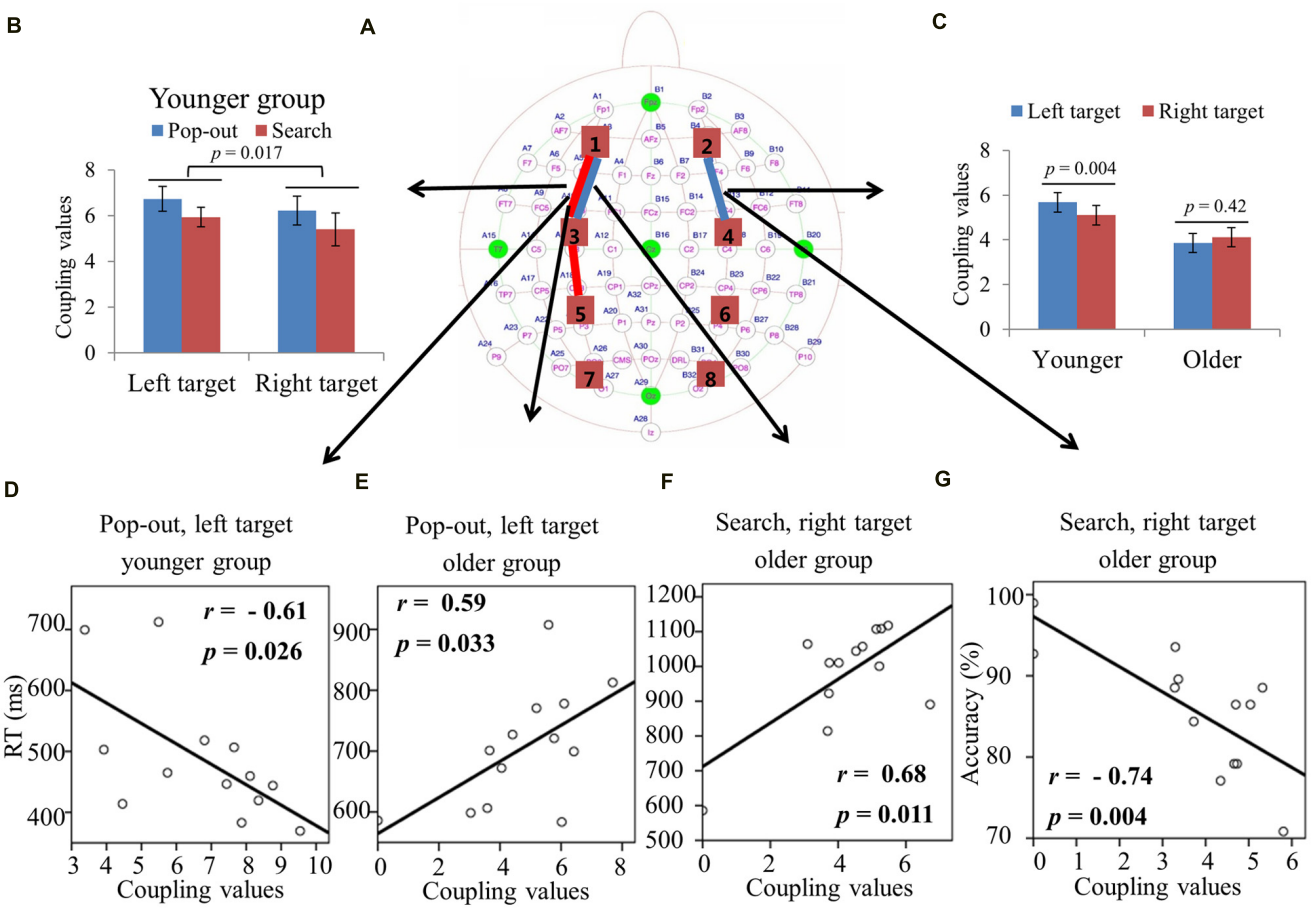
**(A)** Inter-regions coupling in alpha frequency band. Blue lines indicate main effect of age (younger > older) and red line indicates main effect of condition (pop-out > search). **(B)** Coupling values between left prefrontal and central-frontal regions as a function of target field and condition in younger subjects, with greater coupling values in the left field target and in the pop-out condition. There was only a main effect of condition for the older group, with greater coupling values in the pop-out condition. **(C)** Coupling values between right prefrontal and central-frontal regions as a function of target field in younger and older subjects, with greater coupling values in left target than in right target for younger subjects and no difference between right and left targets for old subjects. **(D)** Linear negative regression between mean RT and coupling value between left prefrontal and central-frontal regions for the pop-out condition with the left field target in the younger group. Linear positive regression between mean RT and coupling value between left prefrontal and central-frontal regions for the pop-out condition with the left field target **(E)** and for the search condition with the right target **(F)** in the older group. **(G)** Linear negative regression between mean accuracy and coupling value between right prefrontal and central-frontal regions for the search condition with the right target in the older group.

**Figure 4A** summarizes the main effect of age (blue) and condition (red) among eight regions of interest. As can be seen in **Figure 4A**, there was a main effect of age for coupling values on two long range connections, including the connections between left prefrontal and left central-frontal regions [1 and 3, *F*(1,24) = 5.174, *p* = 0.03], between right prefrontal and right central-frontal regions [2 and 4, *F*(1,24) = 5.87, *p* = 0.02], with smaller coupling values for older subjects. There was a main effect of condition for coupling values on connections between left prefrontal and left central-frontal regions [1 and 3, *F*(1,24) = 14.03, *p* = 0.001], between left central-frontal and left central-parietal regions [3 and 5, *F*(1,24) = 5.87, *p* = 0.02], with smaller coupling values in search condition.

For the connection between left prefrontal and central-frontal regions, there was an interaction between age and target visual field [*F*(1,24) = 9.02, *p* = 0.006]. Hence we divided the test into two repeated measure ANOVA with condition and target visual field as the within-subject factors for younger and older groups, respectively. There was a main effect of target field [*F*(1,12) = 7.75, *p* = 0.017] and condition [*F*(1,12) = 5.09, *p* = 0.044] for younger group, with greater coupling values in the left field target and in the pop-out condition. There was only a main effect of condition [*F*(1,12) = 10.36, *p* = 0.007] for the older group, with greater coupling values in the pop-out condition. **Figure 4B** shows the coupling values in both conditions and target fields for younger group.

For the connection between right prefrontal and central-frontal regions, there was an interaction between age and target visual field [*F*(1,24) = 5.55, *p* = 0.03], and there was no significant effect of condition. Hence we averaged the coupling values of the pop-out and search conditions, and applied repeated measure ANOVA with target visual field (left and right) as the within-subject factor and age (young and old) as the between-subject factor. **Figure 4C** shows the coupling values in both groups for both target fields. There was a main effect of age [*F*(1,24) = 5.87, *p* = 0.02] and an interaction between age and target field [*F*(1,24) = 5.58, *p* = 0.03], with bigger coupling values in left target than in right target for younger subjects [Paried *t*-tests, *t*_(12)_ = 3.58, *p* = 0.004]. Thus, greater bilateral prefronto-frontal coupling were shown in the left visual target for younger group.

**Figure 4D** illustrates the presence of significant negative linear relationship within the younger adults between coupling values of left prefrontal and central-frontal regions and RTs in the pop-out condition with left visual field target [*r* = -0.61, *p* = 0.026]. **Figures 4E,F** illustrate the significant positive linear relationships within the older adults between coupling values of left prefrontal and central-frontal regions and RTs in the pop-out condition with left visual field target [*r* = 0.59, *p* = 0.033] and in the search condition with right visual field target [*r* = 0.68, *p* = 0.011], respectively. **Figure 4G** illustrates the significant negative linear relationship within the older adults between coupling values of right prefrontal and central-frontal regions and accuracies in the search condition with right visual field target [*r* = -0.74, *p* = 0.004]. All other correlation between coupling values and RTs or accuracies was not significant (*p* > 0.05).

#### ROIs Coupling Value in Beta Frequency Band

**FIGURE 5:**
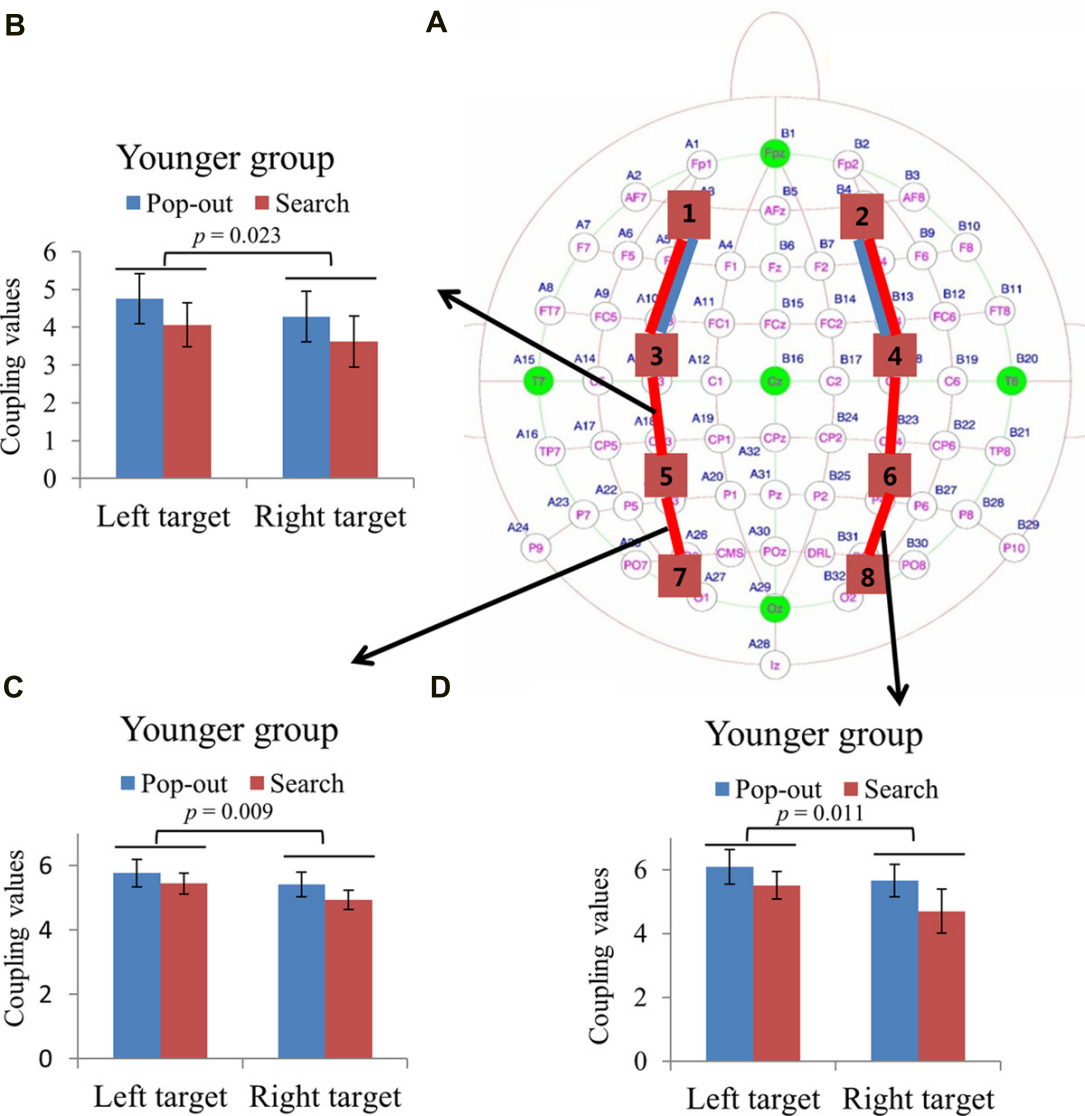
**(A)** Inter-regions coupling in beta frequency band. Blue lines indicate main effect of age (younger > older) and red line indicates main effect of condition (pop-out > search). Coupling values between left central-frontal and central-parietal regions **(B)**, between left central-parietal and parietal-occipital regions **(C)**, and between right central-parietal and parietal-occipital regions **(D)** as a function of target field and condition in younger subjects, with bigger coupling values in left target than in right target, and no such effect was presented in the older group.

**FIGURE 6:**
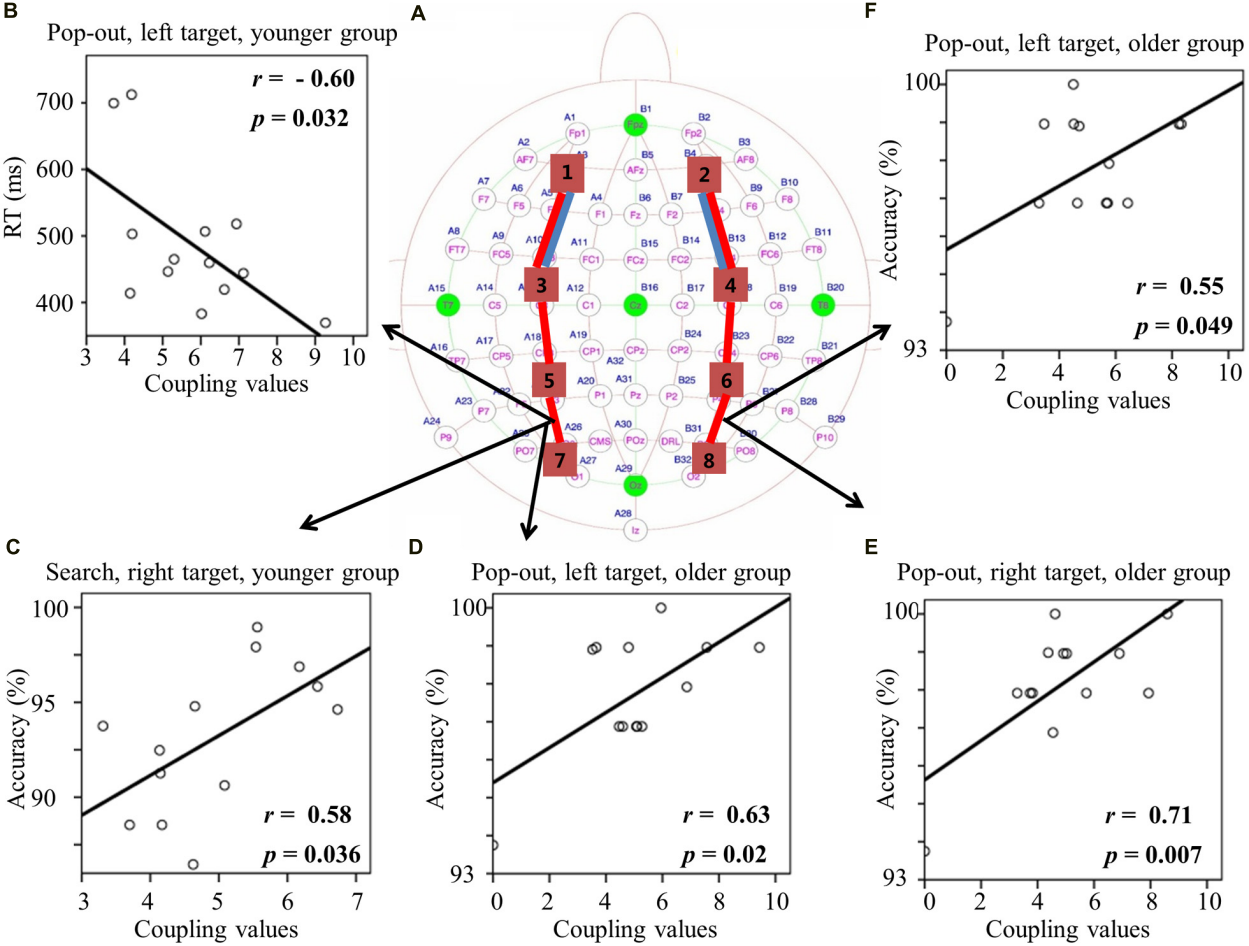
**(A)** Inter-regions coupling in beta frequency band. Blue lines indicate main effect of age (younger > older) and red line indicates main effect of condition (pop-out > search). **(B)** Linear negative regression between mean RT and coupling value between left central-parietal and parietal-occipital regions for the pop-out condition with the left field target in the younger group. Linear positive regression between mean accuracy and coupling value between left central-parietal and parietal-occipital regions for the search condition with the right target in the younger group **(C)** and for the pop-out condition with the left target in the older group **(D)**. Linear positive regression between mean accuracy and coupling value between right central-parietal and parietal-occipital regions for the pop-out condition with the right target **(E)** and the left target **(F)** in the older group.

**Figures 5A and 6A** summarizes the main effect of age and condition among eight regions of interest. As can be seen in **Figure 5A** with blue lines, there was a main effect of age for coupling values on two long range connections, including the connections between left prefrontal and left central-frontal regions [1 and 3, *F*(1,24) = 5.49, *p* = 0.03], between right prefrontal and right central-frontal regions [2 and 4, *F*(1,24) = 5.03, *p* = 0.03], with smaller coupling values for older subjects. In addition, there was a main effect of condition for those two frontal connections [1 and 3, *F*(1,24) = 4.46, *p* = 0.045; 2 and 4, *F*(1,24) = 7.26, *p* = 0.01] marked with red lines in **Figure 5A**, with smaller coupling values in search condition. There was a main effect of condition for coupling values between left central-frontal and left central-parietal regions [3 and 5, *F*(1,24) = 4.87, *p* = 0.04], between right central-frontal and right central-parietal regions [4 and 6, *F*(1,24) = 5.96, *p* = 0.02], between left central-parietal and left parietal-occipital [5 and 7, *F*(1,24) = 5.81, *p* = 0.02], between right central-parietal and right parieto-occipital regions [6 and 8, *F*(1,24) = 7.75, *p* = 0.01], with smaller coupling values in search condition (**Figure 5A**).

Due to the interaction between age and target visual field for these three connections [3 and 5, *F*(1,24) = 4.29, *p* = 0.049; 5 and 7, *F*(1,24) = 7.54, *p* = 0.01; 6 and 8, *F*(1,24) = 4.38, *p* = 0.047], we divided the test into two repeated measure ANOVA with condition and target field as the within-subject factors for both groups, respectively. There was only a main effect of target visual field [3 and 5, *F*(1,12) = 6.73, *p* = 0.023; 5 and 7, *F*(1,12) = 9.69, *p* = 0.009; 6 and 8, *F*(1,12) = 8.93, *p* = 0.011] for the younger group in these three connections, with bigger coupling values in left target than in right target, and no such effect was present for the older group. **Figures 5B–D** show the coupling values in both conditions and target fields for younger group at three connections.

For the coupling values between right prefrontal and central-frontal regions, there were significant positive linear relationships within the older subjects between values and RTs in all four conditions [pop-out and left: *r* = 0.63, *p* = 0.02; pop-out and right: *r* = 0.61, *p* = 0.03; search and left: *r* = 0.58, *p* = 0.04; search and left: *r* = 0.58, *p* = 0.04]. **Figures 6B,C** illustrate the significant positive linear relationships within the younger adults between coupling values of left central-parietal and parietal-occipital regions and performance in the pop-out condition with left target [RTs: *r* = -0.60, *p* = 0.032] and in the search condition with right visual field target [accuracy: *r* = 0.58, *p* = 0.036], respectively. **Figure 6D** illustrates the significant positive linear relationship within the older adults between coupling values of left central-parietal and parietal-occipital regions and accuracies in the pop-out condition with left target [*r* = 0.63, *p* = 0.02]. **Figures 6E,F** illustrate the significant positive linear relationships within the older adults between coupling values of right central-parietal and parietal-occipital regions and accuracies in the pop-out condition with left target [*r* = 0.55, *p* = 0.049] and right target [*r* = 0.71, *p* = 0.007], respectively. All other correlation between coupling values and RTs or accuracies was not significant (*p* > 0.05).

## Discussion

### Aging Effects on Behavior and Inter-regions Coupling

Aging had prominent effects on both behavioral and EEG coupling strength under the control of top–down and bottom–up attention. Aging led to slowed RT and decreased accuracy (**Table 1**), indicating a slowing of cognitive performance with advancing age (Salthouse, 1996). Specifically, greater age-related reductions in accuracy in the search condition than in the pop-out condition were shown, suggesting additional attentional demands for older subjects with increased task complexity (Greenwood et al., 1997; Hommel et al., 2004; Madden et al., 2004, 2007).

Aging was associated with a declined whole-brain coupling strength of theta and alpha frequency bands, with a greater age-related decline in the search than in the pop-out condition (**Table 2**), in accord with their accuracy. Specifically, older adults showed a decreased lateral prefronto-frontal coupling in theta, alpha and beta frequency bands in both conditions (**Figures 3A**, **4A**, and **5A**), which confirmed the well-established age-related decline in allocation of attentional resources efficiency or reduction in inhibitory control functions in attention (Colcombe et al., 2003; Madden et al., 2004; Andrés et al., 2006; Hasher et al., 2008; Lorenzo-López et al., 2008; Gola et al., 2012; Deiber et al., 2013; Gola et al., 2013; Kardos et al., 2014).

Greater prefronto-frontal coupling (left lateral theta coupling, **Figure 3B**; and bilateral alpha coupling, **Figures 4B,C**), greater fronto-parietal coupling (left beta coupling, **Figure 5B**), and greater parieto-occipital coupling (bilateral beta coupling, **Figures 5C,D**) in the left target than in the right target for younger group were presented, suggesting a left visual field advantage (Holländer et al., 2005; Verleger and Smigasiewicz, 2015). This advantage might be related to the right hemispheric dominance in the ventral attentional network, which seems to particularly activate regions largely lateralized to the right hemisphere and involves right temporo-parietal and ventral frontal cortices in healthy subjects (Corbetta and Shulman, 2002; Shulman and Corbetta, 2012; Hong et al., 2015). Left visual field advantage was reduced with aging, supporting the Hemispheric Asymmetry Reduction in Older Adults (HAROLD) theory (Cabeza, 2002). Furthermore, older adults showed a greater left prefronto-frontal theta coupling (right two bars in **Figure 3B**) in the right visual target than in the left visual target, in accord with their quicker RTs, supporting the idea that older adults use bilateral neural circuits as compensation to accomplish visual search tasks (Reuter-Lorenz and Lustig, 2005; Grady, 2008; Reuter-Lorenz and Cappell, 2008). Interestingly, there was a greater left fronto-parietal theta coupling (**Figure 3C**) in the right visual target than in the left visual target for older group, whereas there was no difference for younger group. For younger group, left visual field advantage and the greater activity in the contralateral hemisphere (right visual target) for directing of attention may be counteracting, resulting in no difference between the right and left visual targets. For older group, the contribution of left visual field advantage is reduced, so that the activity in the contralateral hemisphere (right visual target) is in dominance, which is advantageous for task performance. These findings support the idea that right hemispheric dominance in the ventral attentional network is reduced to compensate for the inhibitory dysfunction with aging (Cabeza, 2002).

### Attention Control Effects on Behavior and Inter-regions Coupling

The control of top–down and bottom–up attention had prominent effects on both behavioral and EEG coupling strength. Top–down control led to slowed RT and decreased accuracy for both groups (**Table 1**), indicating that the search task was sufficiently difficult to demand more cognitive effort (Treisman and Gelade, 1980). Top–down attentional control was associated with a declined whole-brain coupling strength of alpha and beta frequency bands, with a smaller coupling in the search than in the pop-out condition (**Table 2**). Specifically, search condition showed a decreased coupling of left fronto-parietal in theta and alpha frequency bands, of left prefronto-frontal in alpha-band, and of six inter-regions in beta-band compared to pop-out condition (**Figures 3A**, **4A**, and **5A**).

Pop-out target detection was mainly associated with greater parieto-occipital beta-coupling strength compared to search condition regardless of aging, in accord with their better performance, which confirmed previous findings that parietal power at beta-band (12–24 Hz) in human and parietal-extrastriate coherences at higher frequency band (35–55 Hz) in monkey were greater in the pop-out than in the search condition (Buschman and Miller, 2007; Li et al., 2010). This supports the idea that posterior parietal cortex is primarily responsible for the encoding of salient stimuli and automatic detection (Constantinidis and Steinmetz, 2005). Fronto-extrastriate coherences at intermediate frequency band (22–34 Hz) were greater in the search than in the pop-out condition (Buschman and Miller, 2007), but in the present study no significant difference in fronto-parietal theta-coupling strength between pop-out and search conditions for young subjects was observed (**Figure 3C**). This may be due to strict statistical method applied for significance in connections when constructing the network, as a result, weaker coupling may be ruled out. For example, if we use the normalized PLV values directly without threshold, a greater theta-coupling strength between left frontal and right occipital regions (regions 3 and 8) in the search than in the pop-out condition in left target for young subjects will be observed [3 and 8, paired *t*-test, *t*_(12)_ = 3.59, *p* = 0.0037]. We prefer to use strict statistical level to decrease the type I error. In summary, our results may indicate that the parieto-occipital coupling of beta-band could serve as a bottom-up function and be vulnerable to top–down attention control in both groups.

### Relationships between Behavior and Inter-regions Coupling

The greater prefronto-frontal coupling showed better performance in theta and alpha bands for younger subjects (**Figures 3D** and **4D**), but worse performance in alpha and beta bands for older subjects (**Figures 4E–G**). These results provided additional information regarding the age-related change in the prefronto-frontal coupling in attentional control during the visual search task, suggesting that prefronto-frontal coupling for both groups may be generated by distinct brain cortices. An fMRI study has reported that younger individuals with higher levels of middle frontal gyrus activation exhibited better performance, and older individuals with higher levels of putamen activation showed worse performance during visual target detection (Madden et al., 2004). In present study, prefronto-frontal coupling results may most reflect connections within frontal gyrus for younger group, and most show connections within deep gray matter structures for older group in compensation for the age-related decline in visual search. Prefronto-frontal coupling was significant as a predictor of behavior for the younger and older groups, but in the opposite direction.

And the bigger parieto-occipital coupling in beta band led to better performance for both groups (**Figures 6B–F**), together with above results, indicating synchrony as a mechanism of attention (Miller and Buschman, 2013). Local synchrony between parieto-occipital cortices in beta-band and between prefronto-frontal cortices in theta, alpha and beta frequency bands may help the brain to improve its signal-to-noise ratio for better processing of bottom–up sensory input and top–down cognitive control, respectively. Parieto-occipital coupling in beta band was significant as a predictor of behavior for the younger and older groups in the same direction, and greater coupling may carry more bottom–up information.

In the current study, we found evidence for age-related changes in the differential roles of fronto-parieto-occipital connectivity at different oscillatory frequencies during the control of top–down and bottom–up attention. Together with evidence from past literature on the animal work on the networks contributing to top–down and bottom–up attention (Buschman and Miller, 2007; Miller and Buschman, 2013), these results suggest that bottom–up and top–down target lead to differential fronto-parieto-occipital connectivity at different oscillatory frequencies in younger and older adults. Greater prefronto-frontal coupling in theta and alpha-bands, fronto-parietal coupling in beta-band, and parieto-occipital coupling in beta-band in the left target than in the right target for younger group indicates a right hemispheric dominance in the ventral attentional network, which is reduced with aging to compensate for the inhibitory dysfunction. While pop-out target detection is mainly associated with greater parieto-occipital beta-coupling strength compared to search condition regardless of aging, in accord with their better performance. Prefronto-frontal coupling in theta, alpha, and beta bands and parieto-occipital coupling in beta band is a predictor of behavior for the both groups. Taken together these findings provide evidence that prefronto-frontal coupling of theta, alpha, and beta frequency bands may serve as a possible basis of aging during visual attention task, while parieto-occipital coupling in beta-band could serve for a bottom–up function and be vulnerable to top–down attention control for younger and older groups.

## Author Contributions

LL: Conceived, designed and performed the experiments. LL and DZ: Analyzed the data. LL: Wrote the paper.

## Conflict of Interest Statement

The authors declare that the research was conducted in the absence of any commercial or financial relationships that could be construed as a potential conflict of interest.

## Supplementary Material

Figure S1 **Linear regression between mean reaction time (RT) and total coupling value for the pop-out condition with the left field target in the older group for theta frequency band**. Each subject is marked as a circle, with 13 circles in total. Pearson correlation coefficient (*r* = 0.588) was significant (*p* = 0.035), indicating a positive linear correlation between RT and total coupling value in this condition.
