# Supplementary figures and images for: Age-Related Inter-Region EEG Coupling Changes During the Control of Bottom–Up and Top–Down Attention
Source: Front Aging Neurosci. 2015 Dec 1;7:223. doi: 10.3389/fnagi.2015.00223 (PMC4664751; doi:10.3389/fnagi.2015.00223)

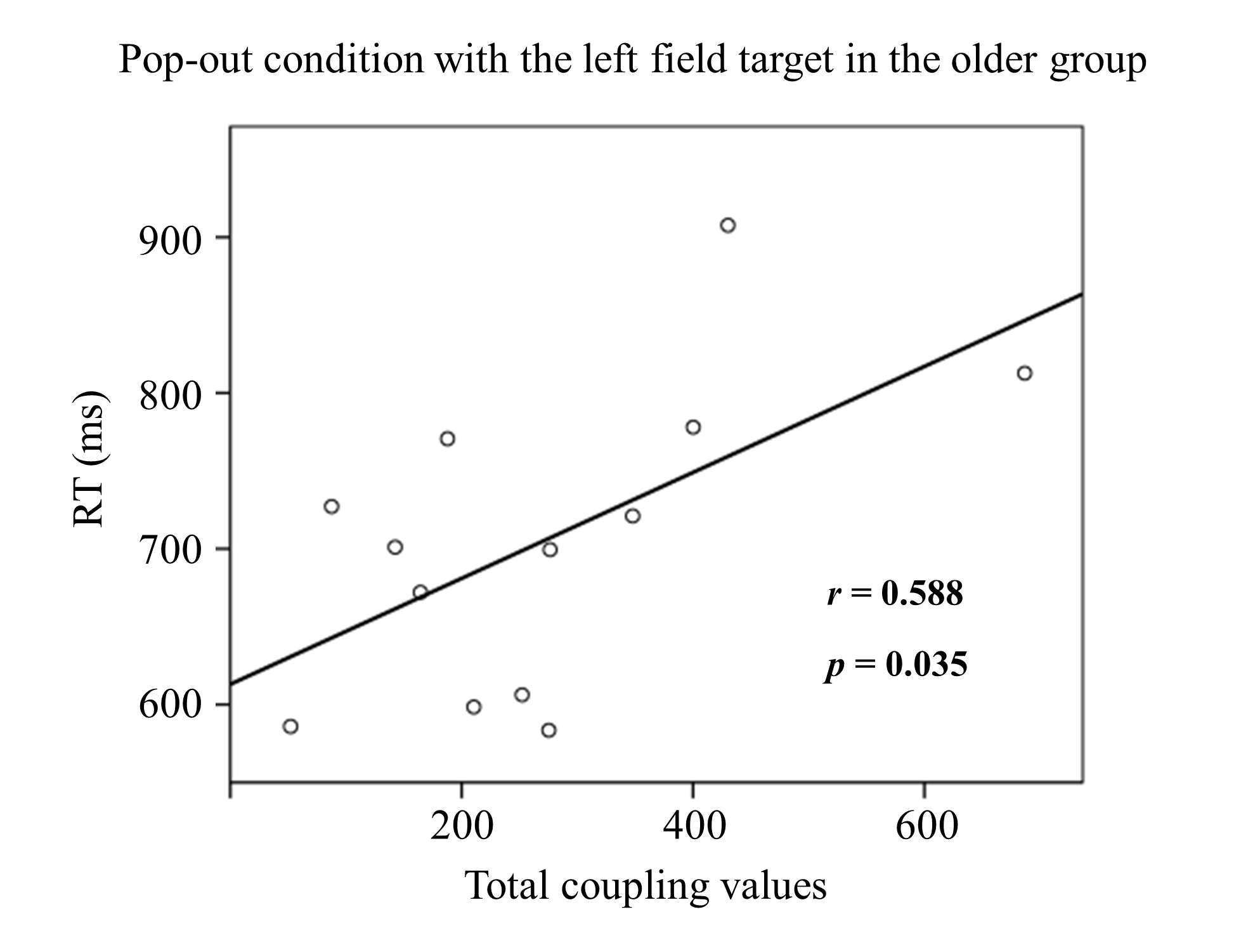

Supplement: Figure S1 — Linear regression between mean reaction time (RT) and total coupling value for the pop-out condition with the left field target in the older group for theta frequency band. Each subject is marked as a circle, with 13 circles in total. Pearson correlation coefficient (r = 0.588) was significant (p = 0.035), indicating a positive linear correlation between RT and total coupling value in this condition. [file Image_1.TIF]
