# Supplementary material for: Age-Related Inter-Region EEG Coupling Changes During the Control of Bottom–Up and Top–Down Attention
Source: Front Aging Neurosci. 2015 Dec 1;7:223. doi: 10.3389/fnagi.2015.00223 (PMC4664751; doi:10.3389/fnagi.2015.00223)
Supplement: Supplementary file 2 [file Table_1.DOCX]

**Table S1.** Significantly statistical effects of three factors of age, condition, and target visual field and interaction on the total coupling values for 9 frequency bands by repeated measure ANOVA.

| Factors | Age | Condition | Target visual field | Age × Target visual field |
| --- | --- | --- | --- | --- |
| Theta band  (4-8 Hz) | √  (p = 0.006) | ×  (p = 0.07) | √  (p = 0.013) | √  (p = 0.018) |
| Alpha band  (8-12 Hz) | √  (p = 0.023) | ×  (p = 0.054) | × | × |
| Beta1 band  (12-16 Hz) | × | √  (p = 0.032) | √  (p = 0.048) | √  (p = 0.015) |
| Beta2 band  (16-20 Hz) | × | √  (p = 0.008) | √  (p = 0.035) | √  (p = 0.039) |
| Beta3 band  (20-24 Hz) | × | √  (p = 0.012) | × | √  (p = 0.026) |
| Beta4 band  (24-28 Hz) | × | √  (p = 0.031) | × | × |
| Beta5 band  (28-32 Hz) | × | √  (p = 0.012) | √  (p = 0.038) | × |
| Gamma1 band  (32-36 Hz) | × | √  (p = 0.009) | × | × |
| Gamma2 band  (36-40 Hz) | × | √  (p = 0.023) | × | × |

√ indicates significant effect (p < 0.05) and × means no significant effect by a repeated measure ANOVA with condition (pop-out and search) and target visual field (left and right) as the within-subject factors and age (young and old) as the between-subject factor for each frequency bands.
